# VeerNet: Using Deep Neural Networks for Curve Classification and Digitization of Raster Well-Log Images

**DOI:** 10.3390/jimaging9070136

**Published:** 2023-07-06

**Authors:** M. Quamer Nasim, Narendra Patwardhan, Tannistha Maiti, Stefano Marrone, Tarry Singh

**Affiliations:** 1 Deepkapha AI Research, Street Vaart ZZ n° 1.d, 9401 GE Assen, The Netherlands; quamer.nasim@deepkapha.com (M.Q.N.); narendraprakash.patwardhan@unina.it (N.P.); tannistha.maiti@deepkapha.com (T.M.); tarry.singh@deepkapha.com (T.S.); 2 Department of Geology and Geophysics, Indian Institute of Technology, Kharagpur 721302, India; 3 Department of Electrical Engineering and Information Technologies (DIETI), University of Naples Federico II, Via Claudio 21, 80125 Naples, Italy

**Keywords:** raster log, digitization, transformer, deep learning, well-log curves

## Abstract

Raster logs are scanned representations of the analog data recorded in subsurface drilling. Geologists rely on these images to interpret well-log curves and deduce the physical properties of geological formations. Scanned images contain various artifacts, including hand-written texts, brightness variability, scan defects, etc. The manual effort involved in reading the data is substantial. To mitigate this, unsupervised computer vision techniques are employed to extract and interpret the curves digitally. Existing algorithms predominantly require manual intervention, resulting in slow processing times, and are erroneous. This research aims to address these challenges by proposing VeerNet, a deep neural network architecture designed to semantically segment the raster images from the background grid to classify and digitize (i.e., extracting the analytic formulation of the written curve) the well-log data. The proposed approach is based on a modified UNet-inspired architecture leveraging an attention-augmented read–process–write strategy to balance retaining key signals while dealing with the different input–output sizes. The reported results show that the proposed architecture efficiently classifies and digitizes the curves with an overall F1 score of 35% and Intersection over Union of 30%, achieving 97% recall and 0.11 Mean Absolute Error when compared with real data on binary segmentation of multiple curves. Finally, we analyzed VeerNet’s ability in predicting Gamma-ray values, achieving a Pearson coefficient score of 0.62 when compared to measured data.

## 1. Introduction

Development or production wells are drilled specifically for extracting oil or gas from fields that have been proven to have economic viability and recoverable reserves. Well-logging is the process of taking measurements of various rock properties along the length of the well down into the ground by drilling tools. The digital log curves are functions of lithology, porosity, fluid content, and textural variation of formation. The well-logging parameters are used to derive lithofacies groups and facies-by-facies descriptions of rock properties. Before the advent of digital logging instruments, well-logging data were drawn on the parameter graph in curve format. Well-logging parameter graphs have many disadvantages: large size, ample memory space, and interference like gridlines. Therefore, it is necessary to convert well-logging parameter graphs into X-Y coordinates, where X represents parameter values and Y represents depth values. Raster logs are scanned copies of paper logs saved as image files. Well-log data saved as depth-calibrated raster images provide an economical alternative to digital formats for preserving this valuable information in the future [1]. Although often discarded after vectorization, raster imaged well-logs may be the key to a global computer-readable format for legacy hardcopy data. These legacy data are stored on multiple media and contains information for various applications in addition to resource exploration and development, such as environmental protection, water management, global change studies, and primary and applied research. A raster log needs to be digitized using software, and digitized data are used for geological subsurface modeling [2].

**Figure 1:**
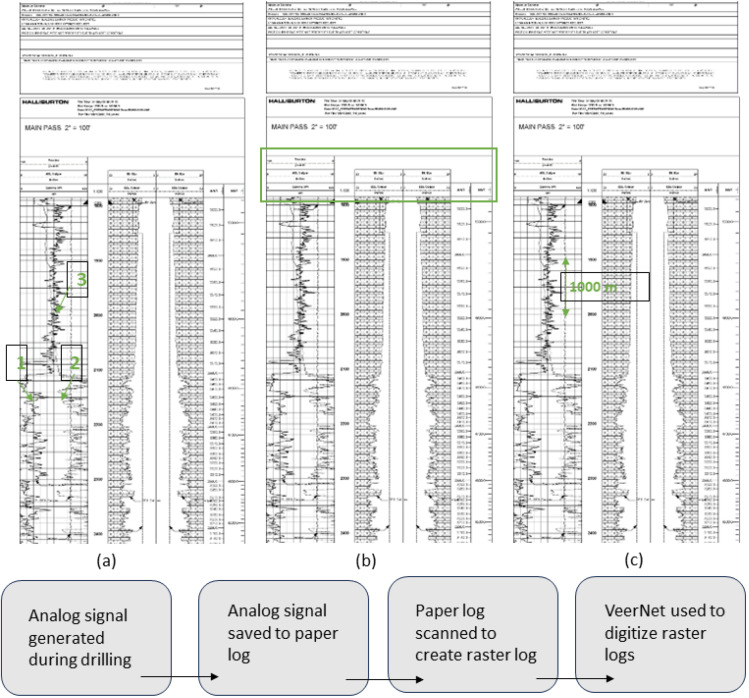
(**a**) 1,2,3 logs corresponding to GR, Caliper, and tension. (**b**) Header section showing the scales, of curves (**c**) depth line coding for 5′ log corresponding to 1000 m. Bottom: Flow chart showing the proposed approach.

Well-logs are the primary means of determining formation depth and oil in place. A log records information about the geological formations that were drilled. There are many types of logs; each is used to determine specific information about the subsurface. Various tools, such as electrical, radioactive, or acoustic, are used to take downhole measurements, where an electrical cable and winch are lowered downhole, and the signal from the tool is transmitted, received, and processed by displaying graphs on a computer that geologists and engineers can interpret to determine the depths and thickness of formations of interest, contour map of the formation, etc. [3]. In the pre-digital (1930–1970) era, energy professionals faced the problem of making profitable use of wireline well-log data, as they were recorded as paper prints (analog), which are known as raster logs [4] (Figure 1). Up until the 1970s, all well-log recordings were made using analog systems. The logs were made on paper with ink or a light beam on photographic film. All the recordings were made in real time. There were no recordings made for later playback. All these raster logs are archived today in back room filing cabinets and/or on microfiche. The lost art of extracting vital petrophysical “answers” from single-point readings taken off wavy curved lines on a paper print can be quite daunting to those schooled in modern digital well-log recordings and continuous data processing.

A typical raster log includes the header, which provides specific information about the well, such as the operating company, well location information, and type of log run. Even the very oldest log print is the standardized API log grid (see Figure 1). It consists of three “tracks”, each 21/2 inch wide, with a “depth track”, half an inch wide, between the first (left-hand) track and the other two, conventionally known as “Tracks 2 and 3”. The main log section, or the graph, charts the depth reached vertically; the horizontal scale is the measurement scale, which can be represented linearly or by logarithms. Inserts are found throughout the graph at each major log section, identifying each curve. Curves on the log, also called traces, readings, or measurements, can be represented by solid, long-dashed, short-dashed, or dotted lines to decipher between the different measurements on the log. The final part of the log includes the tool calibrations before and after the log was conducted, ensuring that the log is accurate. Much of the difficulty of dealing with old analog prints arises from the scaling of the curves. Thus, a large part of the process of making any log analysis is wrapped up in things. Hence, it is essential to create software that is scale-agnostic.

In summary, the existing commercial raster log digitizers capture data from raster logs by scanning the paper log and using image processing algorithms to identify and extract relevant data points. However, existing software requires continuous manual intervention, thus resulting in extremely time-consuming operations. In the present study, we propose a novel transformer-based deep learning model named VeerNet, which employs self-attention mechanisms to identify individual curves from a single track of raster paper logs. The straightforward design of the digitizer system requires only four steps to convert raster images to digital values. The easy-to-use approach can significantly reduce time and increase accuracy. For the very first time, a codebase on deep-learning raster log digitization is made publicly available so others can review and improve it, and the code can become a valuable resource for the community.

The rest of the paper is organized as follows: Section 2 reviews the relevant literature on the previous work completed by researchers to extract values from the log curves and differentiate between grid and curve values; Section 3 discusses VeerNet, our proposed architecture, in detail; Section 4 describes the considered experimental setup, including the used dataset, training parameters, and considered loss functions; Section 5 describes the results generated from VeerNet and real data; Section 6 discusses the results and limitations; finally, Section 7 offers conclusions and suggestions for future research.

## 2. Previous Work

The commercially used logging curve digitization software, Neuralog, is based on Scanning, Compressing, Tracing, and Rectifying (SCTR). However, due to the interference of the background grid, this software frequently pauses during curve tracking. Several unsupervised computer vision methods have been implemented to digitize the log data embedded in the binary image. Data are usually interpolated in these situations to, in comparison, the original data [5]. Well-log digitization could be performed through two kinds of approaches: pixel-based methods and non-pixel-based methods. Pixel-based methods include the thinning process and the Global Curve Vectorization (GCV) method [6,7,8,9]. The thinning method reduces the width of a line to only one pixel, leaving only the skeleton that can characterize its features. The main disadvantage of the thinning process is that it has a high time complexity, loses line width information, and is prone to deformation and wrong branches in the intersection area. The GCV method is suitable for line processing but poor for point line processing [10].

Non-pixel-based methods mainly fall into two categories: contour-based and adjacency-graph-based. The contour-based approach [11] extracts the contour of the image first and then finds the matched contour pairs. The adjacency graph method first applies run-length encoding to graphs, then analyzes the segments and generates various adjacency graph structures, such as Line Adjacency Graph (LAG) [12] and Block Adjacency Graph (BAG) [13,14]. Ref. [15] used the SCTR approach by employing the LAG data structure. Ref. [16] improved the SCTR method and put forward the Preprocessing, Compressing, Tracing, and Rectifying (PCTR) method. Ref. [10] proposed an algorithm for erasing grid lines and reconstructing strokes in Chinese handwriting based on BAG. However, these methods have limitations when analyzing complex situations in well-logging parameter graphs, especially the analysis of nodes. Ref. [10] used morphological image processing and the pixel statistics method to eliminate gridlines, isolating the curves and the gridlines. Then, the remaining grid lines and noise points are cleared according to the characteristics of the small size of their connected components. However, all these existing methods need manual intervention, which is not the desired option, especially when the paper logs have a huge size of >10 MB. Recently, deep-learning-based image segmentation has been used to extract metadata from the log header and has been proposed by Laxman Katole et al. [17]. The method employs a two-stage Conditional Generative Adversarial Network (cGAN) that extracts the curve pixels from the plot segments.

## 3. Proposed Approach

The resolution of raster logs is significantly higher compared to images used in traditional image segmentation pipelines. However, this poses limitations on transfer learning due to memory requirements. To address this issue, we propose *VeerNet*, a modified UNet-inspired architecture that strikes a balance between preserving key signals and reducing dimensionality.

**Figure 2:**
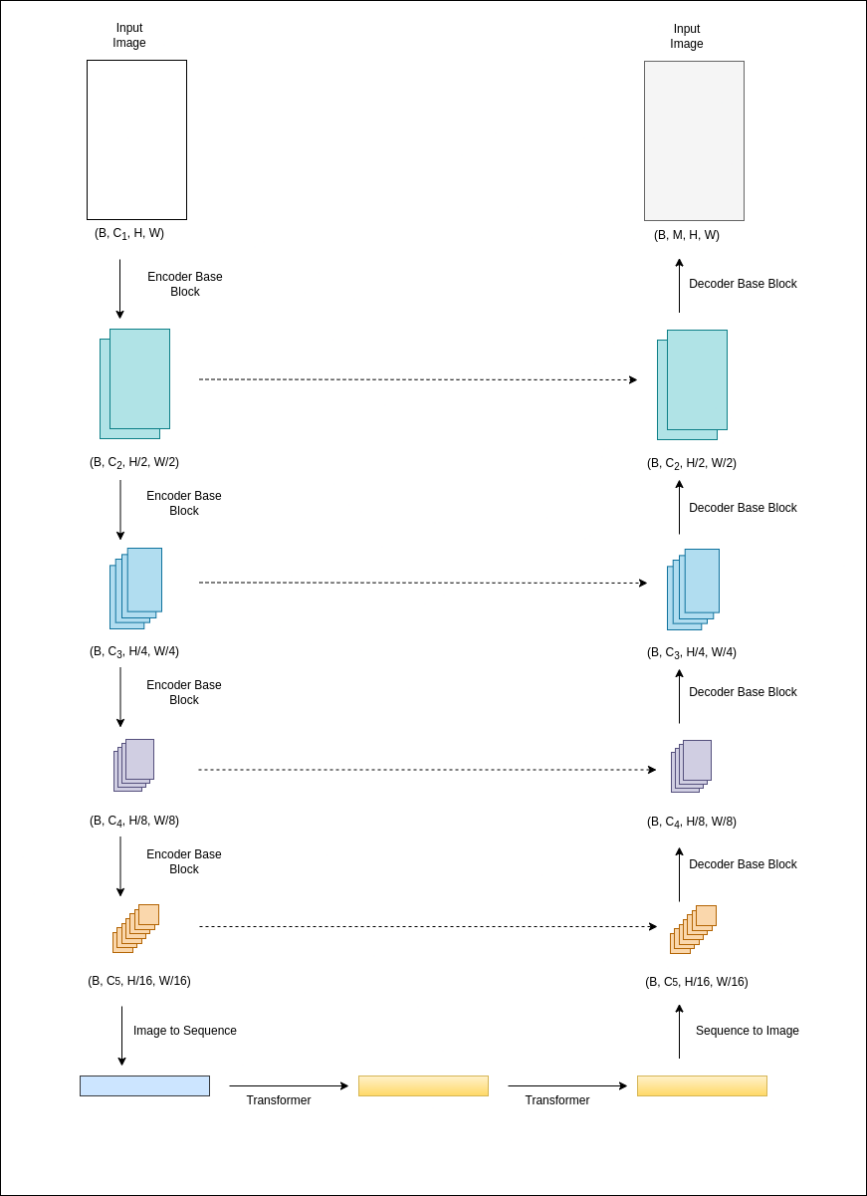
The proposed transformer-augmented U-Net.

VeerNet utilizes an attention-augmented read–process–write architecture. In the encoder, the input undergoes downsampling through a series of four blocks comprising 2D convolutions, which reduce spatial resolution by a factor of 2, followed by group normalization and GELU non-linearity. The transformer blocks the process and refines the internal representation learned by the encoder [18]. The sequence is then reformed to match the encoder-level resolutions with a decoder. The decoder blocks consist of bilinear upsampling, 2D convolution, group normalization, and RELU activation. Residual connections enhance the signal strength at each decoder level (shown in Figure 2). The final decoder block generates spatial masks, indicating the presence of corresponding log curves. This approach combines the strengths of both UNet and modern transformer-based models, enabling efficient downsampling and learning rich, global context-aware representations using transformers.

Our model takes the user’s paper log as input and applies filters to extract feature maps. Subsequently, the model reduces the feature map size of the scanned paper log. The middle layer focuses on the log signals while disregarding grid lines and annotations. This derived information is used to restore the log feature map to its original shape, effectively removing everything except the signals from the image. Consequently, we obtain an image containing only the desired signals without any grid lines or other noise. From this signal image or mask, we extract a 1D signal and save it in CSV/LAS files.

The VeerNet model follows an encoder–decoder architecture. The encoder consists of residual blocks, allowing information flow from shallower to deeper layers. Five residual blocks encode the image into a feature map that is 1/32th the size of the original image. Following the encoder, two transformer layers, each comprising an attention layer, are employed. These attention layers compute weights for the input feature map, producing an output vector that encodes information on how each pixel should attend to all other pixels in the image. Finally, five decoder layers, each including an upscaling operation and a convolution operation, are used to achieve the exact output size as given in the input. The architecture consistently produces masks of the same size as the original image. Post-processing steps are then applied to the predicted masks to generate CSV files, which contain the digitized values of the well-log curve(s).

## 4. Experimental Setup

We trained on a dataset of 10,000 images. The images consist of a single track that can either have two or three well-log curves generated from LAS and raster image files obtained from Texas RRC data (https://www.rrc.state.tx.us/, accessed on 10 March 2022).

The well-log curves used are: gamma ray, calliper, SP, shallow resistivity, medium resistivity, deep resistivity, neutron porosity, and density logs. First, we generated mini-batches from the extracted well-log curves of LAS files. Then, a Gaussian process regression fit was applied to the mini-batches, which generated about 100 distributions for the curves. Next, the distribution was randomly selected and sampled. Finally, we created the dataset with data sampled from the distribution. To treat the NaN values in the LAS files, we implemented two methods, i.e., (a) fill the NaN value with a constant number and (b) remove the NaN values. A detailed analysis of the real dataset is available in Table 1.

We performed several experiments with various loss functions, such as Dice, Tversky, Lovaz, Focal, and Sparse Cross Entropy (SCE) (Table 2), considering two performance metrics, i.e., $Pearsoncoefficient(r_{p})$ and *p*-value (Table 3), and also changing the number of transformers (i.e., 4, 5, and 6). In all the experiments, the maximum number of epochs to train the models was set to 250, while the learning rate varies from 1.5 $\times \;10^{-3}$ to 2.5 $\times \;10^{-3}$. We used a cosine learning rate for the learning rate scheduler for all the runs.

In our study, we utilized Python 3.8.12 and PyTorch 1.13.0 as the software tools for implementing and evaluating our approach. The hardware setup consisted of five workers, each equipped with six AMD CPU cores and one NVIDIA A100-SXM-80GB GPU. With a total of 512 GB of RAM, we ensured efficient processing of large datasets and memory-intensive operations.

## 5. Results

We treat this problem in two stages: (a) a 2multi-class classification problem to identify the well-log curves from the background and separate them into individual curves and (b) a regression analysis of individual curves to find the goodness of fit. In both cases, 80% of the total instance were kept for training, while 20% of them were used for validation based on the results reported.

The study’s central hypothesis is based on a low signal/noise ratio; while efforts were made to introduce noise similar to that observed in raster paper logs, the technique still has limitations. VeerNet achieves an overall F1 score of >35% (Table 4), while the maximum Intersection over Union (IoU) score is >30%. In the first stage of the evaluation of the mask, the performance metrics used were IoU and F1 Score. IoU is a metric that evaluates deep learning algorithms by estimating how well a predicted mask matches the ground truth data. The F1 score sums up the predictive performance of a model by combining two otherwise competing metrics—precision and recall. The experiments were performed with five different loss functions, with Lovasz loss performing best among all the loss functions.

We performed two different segmentation tasks: (i) Binary segmentation and (ii) multi-class segmentation. Binary segmentation has three binary masks as ground truth, one for each curve. Multi-class segmentation has three classes in each mask: 0, 1, 2, i.e., background, curve 1, and curve 2, respectively. Once the masks are generated, a post-processing step removes disconnected pixels that are not part of the actual signal. Experiments were performed with a default learning rate (refer to Table 4). We used the number of transformers as a hyperparameter to study the effect on F1 scores. No significant metrics change was observed. The study on the impact of the number of transformers is inconclusive. SCE and Dice 3 transformers obtain better F1 scores than 5, 4, and 6, whereas for Tversky, using six transformers provided a better score. Lovasz and SCE loss displayed the best results (see Table 4).

**Figure 3:**
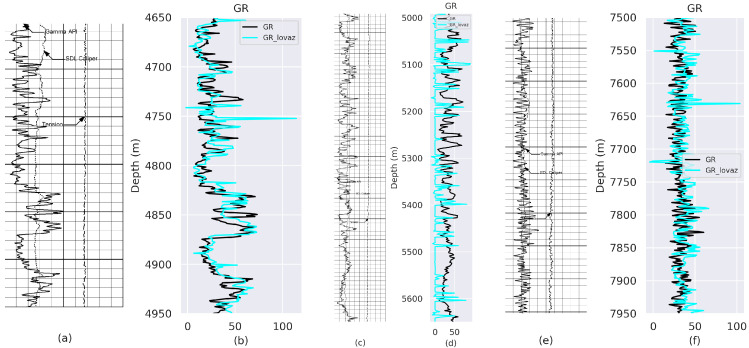
Comparison between the ground truth and prediction from the model trained using Lovaz Loss. (**a**) Rsater Log-Depth 4700 to 5000 m; (**c**) Rsater Log-Depth 4990 to 5650 m; (**e**) Rsater Log-Depth 7500 to 7950 m. (**b**,**d**,**f**) represent the fit between Ground Truth (GT) and predicted GR values for (**a**–**c**), respectively.

**Figure 4:**
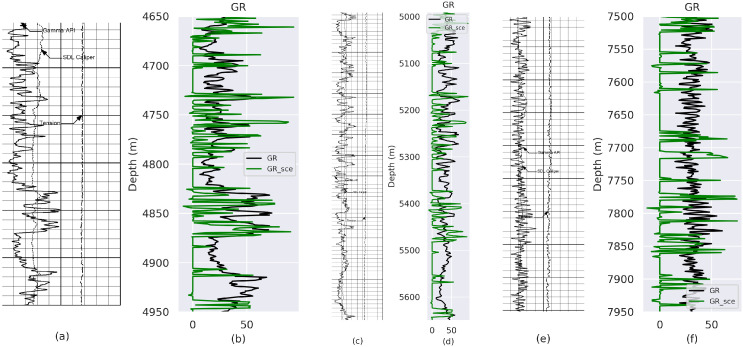
Comparison between GT and prediction from model trained using SCE Loss. (**a**) Rsater Log-Depth 4700 to 5000 m; (**c**) Rsater Log-Depth 4990 to 5650 m; (**e**) Rsater Log-Depth 7500 to 7950 m. (**b**,**d**,**f**) represent the fit between GT and predicted GR values for (**a**–**c**), respectively.

**Figure 5:**
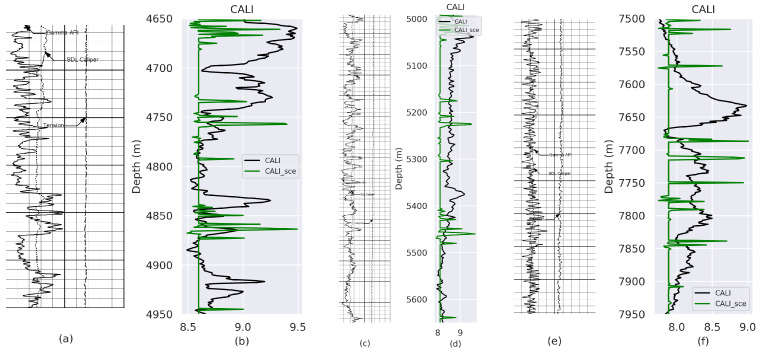
Comparison between GT and Prediction from model trained using SCE Loss. (**a**) Rsater Log-Depth 4700 to 5000 m; (**c**) Rsater Log-Depth 4990 to 5650 m; (**e**) Rsater Log-Depth 7500 to 7950 m. (**b**,**d**,**f**) represent the fit between GT and predicted CALI values for (**a**–**c**), respectively.

**Figure 6:**
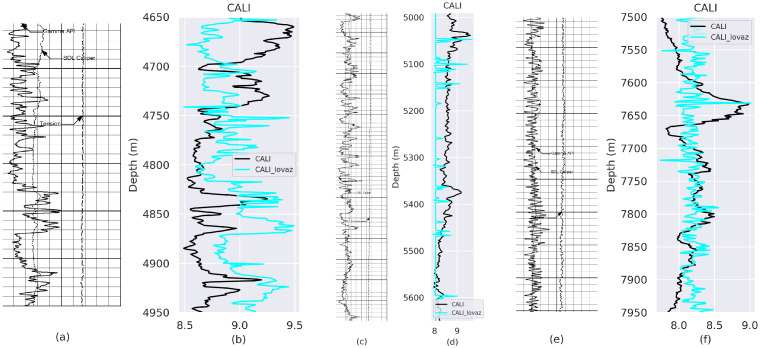
Comparison between GT and prediction from model trained using Lovaz Loss. (**a**) Rsater Log-Depth 4700 to 5000 m; (**c**) Rsater Log-Depth 4990 to 5650 m; (**e**) Rsater Log-Depth 7500 to 7950 m. (**b**,**d**,**f**) represent the fit between GT and predicted CALI values for (**a**–**c**), respectively.

Experimental results obtained from VeerNet models on Gamma Ray (GR) and Caliper (CALI) from well with API number: 42-165-369222 and well name: UNIVERSITY 6-13 No. 1 are listed in Figure 3, Figure 4, Figure 5 and Figure 6, which demonstrate that the proposed model fits the actual well-log curve with a certain goodness of fit. To experimentally explore the potential fit of the digitized data with native LAS data, we implemented two model variants with loss, i.e., Lovasz and SCE.

In statistical studies, the null hypothesis is a default hypothesis where the quantity (typically the difference between two situations) to be measured is zero (null). In this scenario, the null hypothesis is to determine if there is an indication that the calculated and original samples are derived from different distributions. The *p*-value is the probability of obtaining test results at least as extreme as the results observed, assuming that the null hypothesis is correct. A *p*-value < 0.05 is sufficient to reject the null hypothesis and conclude that a significant difference between the two distributions does exist. To measure the similarity derived GR from Lovasz loss and SCE loss, we determine that the $r_{p}$ value and *p*-value for the model with Lovasz loss and SCE for GR (Table 5) are high, indicating that the derived value and the native LAS can correlate. Furthermore, the *p*-value < 0.05; hence, the distributions are statistically significant, and therefore, the null hypothesis is rejected. To measure the similarity derived for the CALI log from Lovasz loss and SCE loss, we calculate the $r_{p}$ value and *p*-value. The $r_{p}$ value for the model with Lovasz loss has a negative correlation. In contrast, for SCE, a low $r_{p}$ value of 0.12 is obtained (Table 5), indicating that the derived value and the native LAS value remain inconclusive. Furthermore, the *p*-value < 0.05 is observed for both model variants with Lovasz and SCE; hence, the distributions are statistically significant, and therefore, the null hypothesis is rejected.

**Figure 7:**
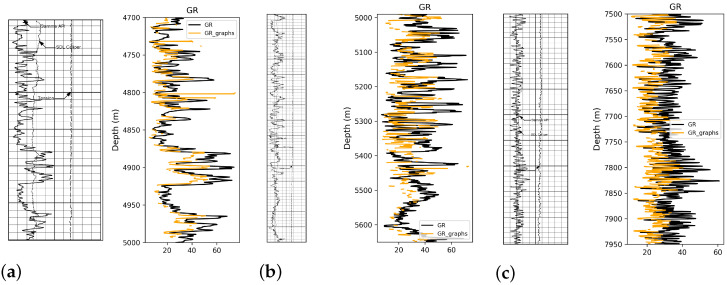
Comparison between native LAS Gamma Ray and the graph-based method with corresponding depth sections of the raster log image. (**a**) Depth 4700 to 5000 m; (**b**) Depth 4990 to 5650 m; (**c**) 7500 to 7950 m.

We compare our results with previous techniques proposed by Yuan and Yang [10]. The proposed graph-based gridline approach consists of two steps: in the first step, eliminate vertical grid lines by dilation based on structuring element followed by the pixel statistics method to remove horizontal gridlines. In the pixel statistics method, we count the number of black pixels per line of the well-logging graphs, denoted by the counter. If the counter is less than the threshold, it is a well-log curve. Otherwise, it is a horizontal gridline, and the horizontal gridline will be removed. The major drawback of this technique is that it can only digitize one curve at a time. The results are presented in Figure 7; while the vertical and horizontal lines are eliminated from the well-log track, the technique also removes the CALI logs. The method is also dependent on the threshold values for pixel statistics.

## 6. Discussions

In this study, we proposed VeerNet, a deep learning model for digitizing raster well-log images. The model achieved satisfactory results in classifying well-log curves and separating them from the background grid, with an average classification accuracy of over 35%. The experimental results demonstrated the effectiveness of VeerNet in generating digitized GR and CALI values. The comparison between the derived values and the native LAS data showed a significant correlation for the GR log. We also explored different loss functions and variations in the number of transformers used in VeerNet. Among the tested loss functions, Lovasz loss and SCE loss yielded the best results in terms of the F1 score. Although efforts were made to introduce noise similar to that observed in raster paper logs, the technique still has its constraints. The low signal-to-noise ratio remains a challenge, which can affect the accuracy of the digitized values. Overall, VeerNet provides a promising solution for digitizing raster well-log images.

One of the reasons for CALI logs not performing well is their skewed distribution. A larger dataset for CALI from diverse geology is required to improve the model’s accuracy. The model is not trained on a specific reservoir; we want to keep it generic. For future studies, we will include data from geologic reservoirs and inspect the performance on logs such as resistivity, sonic, etc.

Another limitation of this study is when using one track, the user needs to provide a cropped section from the front end. This functionality could include an error in manual analysis in providing the scale value. To overcome these difficulties, we propose (1) keeping all three or four tracks of the raster well-log images and (2) designing Optical Character Recognition (OCR) architecture to automatically read scales of the well-log curves rather than providing them manually. In the next iteration of VeerNet, we will provide a detailed analysis of all the hyperparameters discussed above.

While we acknowledge that some of the accuracy scores obtained in our experiments may not be ideal, it is important to note that this is primarily due to the lack of large open-sourced datasets available for training our model. Unfortunately, the limited availability of such data adversely affects the overall performance. However, we firmly believe that our proposed approach is still a valuable and promising solution. We believe that this paper serves as a gateway to a new and improved method/framework for digitizing well-logs, offering the potential for significant developments in the field.

## 7. Conclusions

In this study, we have addressed the significance of scanned raster logs and their crucial role in geological reservoir studies. We have provided an overview of the importance of digitizing raster logs and discussed relevant software. Additionally, we have explored previous studies and algorithms pertaining to the digitization of raster logs.

To train our deep learning models, we have presented the methodology for generating synthetic and real data. We have thoroughly described the experimental setup, including the components of our proposed deep learning model, VeerNet. Moreover, we have made our code base publicly available, aiming to contribute to the wider research community.

Our proposed solution for digitizing raster well-log images offers a simplified and less manual approach compared to existing techniques. The solution is not only efficient but also scalable. VeerNet has demonstrated remarkable proficiency in classifying well-log curves, achieving an average classification accuracy exceeding 35%. This model effectively identifies well-log curves from the background grid, surpassing the capabilities of current technology.

Furthermore, we have showcased the digitized GR values obtained from various sections of the well, highlighting their close alignment with native las data, as indicated by a high Pearson coefficient of 0.62. This correlation demonstrates a substantial improvement in the overall goodness of fit to real data.

As this study represents a novel approach, no baseline model exists for a direct comparison of results. However, we have introduced innovative techniques for generating synthetic well-log data using Gaussian process regression, ensuring basin-independent synthetic data generation.

Unlike traditional well-log digitization software that often encounters interference with the background grid and experiences frequent pauses during curve tracking, VeerNet provides a rapid and easily deployable solution. It streamlines the process into four simple steps: uploading scanned paper logs, selecting the desired section for cropping, verifying the scales and range values of curves, and finally saving the digitized values.

In conclusion, this study emphasizes the significance of scanned raster logs and introduces VeerNet as a robust solution for digitizing raster well-log images. Our research contributes to the field by offering an efficient, scalable, and accurate approach to well-log digitization, paving the way for improved geological reservoir studies.

## Figures and Tables

**Table 1:** Distribution of images based on two/three tracks and the respective well-log curves used in model training and validation. The dataset is generated from processed data.

Track	Well-Log Curve	Total Number of Curves in Each Track	Number of Images Generated
Track 1	SP, Gamma Ray, Caliper	1	1600
		2	1600
		3	1600
Track 2	Shallow Resistivity, Medium Resistivity and Deep Resistivity	1	1408
		2	1280
		3	1280
Track 3	Density log and Neutron Porosity Log	1	1280
		2	1280

**Table 2:** Loss function used in hyperparameter tuning.

Loss Functions	Description
Dice Loss	Dice Loss optimizes networks based on the dice overlap coefficient between the predicted segmentation result and the ground truth annotation. Thus, it can effectively alleviate the imbalance between the foreground and background [19].
Tversky Loss	Adjusting the Tversky similarity index [20] allows placing emphasis on false negatives in training a network that generalizes and performs well in highly imbalanced data, as it leads to high sensitivity.
Lovasz Loss	The Lovasz loss achieves direct optimization of the mean intersection-over-union loss [21]. It improves the IoU.
Focal Loss	Focal Loss focuses on learning hard misclassified examples. $(1-p_{t})\gamma$ is added to the cross-entropy loss, with a tunable focusing parameter γ≥0.
SCE Loss	SCE Loss is used when the classes are mutually exclusive (e.g., when each sample belongs precisely to one class) [22]. It works on integers, but they must be class indices, not actual values. The loss computes logarithm only for the output index, which ground truth indicates. Thus, when the model output is, for example, [0.1, 0.3, 0.7] and the ground truth is 3 (if indexed from 1), the loss computes the only logarithm of 0.7. It calculates the logarithm once per instance and omits the summation, which leads to better performance.

**Table 3:** Evaluation matrix and description of parameters.

Evaluation Matrix Parameter	Description
Pearson Coefficient ($r_{p}$)	The Pearson Coefficient measures linear correlation between two variables X and Y for finite sample sizes [23]. It measures the strength of the association between two variables and the direction of their relationship. The strength of the relationship is determined by the value of the correlation.
*p*-value	The *p* value estimates the linear relationship between two variables. In this study, a *p*-value < 0.05 refers to a statistically significant difference between variables and supports that two samples did not come from the same distribution. When a *p*-value indicates no statistically significant difference, the two samples originate from the same distribution.

**Table 4:** Results of VeerNet based on different hyperparameters. The numbers in the braces indicate the results obtained by training for the different number of transformers.

Loss Function and Transformer Numbers	F1	IoU
Lovaz, 5	0.35	0.30
SCE, 3 (4,6)	0.34 (0.33, 0.33)	0.30 (0.30, 0.29)
Focal, 6	0.32	0.28
Tversky, 6 (4)	0.24 (0.22)	0.20 (0.22)
Dice, 4 (5)	0.25 (0.23)	0.22 (0.19)

**Table 5:** Statistical variants for VeerNet. The $r_{p}$ value and *p*-value determine how well VeerNet performed when compared to real data.

Loss Function and Curve Specification	Pearson Coefficient	*p*-Value
Lovasz and GR	0.62	0.0
SCE and GR	0.27	0.0
Lovaz and CALI	−0.21	0.0
SCE and CALI	0.12	0.04

## Data Availability

All the data and code will be made available after the publication of the paper.

## Abbreviations

The following abbreviations are used in this manuscript:

MDPI	Multidisciplinary Digital Publishing Institute
DOAJ	Directory of open access journals
TLA	Three-letter acronym
LD	Linear dichroism
